# How should we measure proportionality on relative gene expression data?

**DOI:** 10.1007/s12064-015-0220-8

**Published:** 2016-01-13

**Authors:** Ionas Erb, Cedric Notredame

**Affiliations:** Centre for Genomic Regulation (CRG), The Barcelona Institute of Science and Technology, Dr Aiguader 88, 08003 Barcelona, Spain; Universitat Pompeu Fabra (UPF), Barcelona, Spain

**Keywords:** Co-expression, Data normalization, Gene networks, Spurious correlation, Log-ratio analysis, Compositional data

## Abstract

Correlation is ubiquitously used in gene expression analysis although its validity as an objective criterion is often questionable. If no normalization reflecting the original mRNA counts in the cells is available, correlation between genes becomes spurious. Yet the need for normalization can be bypassed using a relative analysis approach called *log-ratio analysis*. This approach can be used to identify *proportional* gene pairs, i.e. a subset of pairs whose correlation can be inferred correctly from unnormalized data due to their vanishing log-ratio variance. To interpret the size of non-zero log-ratio variances, a proposal for a scaling with respect to the variance of one member of the gene pair was recently made by Lovell et al. Here we derive analytically how spurious proportionality is introduced when using a scaling. We base our analysis on a symmetric proportionality coefficient (briefly mentioned in Lovell et al.) that has a number of advantages over their statistic. We show in detail how the choice of reference needed for the scaling determines which gene pairs are identified as proportional. We demonstrate that using an unchanged gene as a reference has huge advantages in terms of sensitivity. We also explore the link between proportionality and partial correlation and derive expressions for a partial proportionality coefficient. A brief data-analysis part puts the discussed concepts into practice.

## Introduction

The frequently compositional nature of biological data and its methodological implications (a.k.a. analysis of “closed” data) have not been widely acknowledged yet Lovell et al. (2011). One prominent example is RNA sequencing data, where the final readouts (total number of sequenced reads per library) need to be multiplied by factors depending on the library to recover the original total amounts of mRNA. This exact information about absolute quantities of mRNA can be unavailable or hard to precisely estimate - depending on the chosen protocols. As a result, assumptions need to be made with respect to parameter stability across experiments that will necessarily influence the final interpretation. For instance, most protocols assume equality of total mRNA amounts (in the cells) across samples, even though this (rather strong) assumption can well be violated Lovén et al. (2012) and lead to inaccurate conclusions. If total mRNA amounts of origin are not the same between samples, the dominance of certain transcripts in one condition can lead to other transcripts yielding lower read percentages even though their totals remain unchanged between conditions. This problem has been addressed by “effective library size” normalization Robinson and Oshlack (2012), a method that needs the weaker (and most parsimonious) assumption that most genes between samples remain unchanged and thus retain comparable expression levels. Also here we can think of many situations where this is not true, and there is simply no “magic powder that can be sprinkled on closed data to make them open” Aitchison (2003). In data constrained to constant sums (e.g. RNA-seq data constrained to a fixed number of reads) individual readouts are not fully independent, and their comparison can easily be confounded, especially when drawing correlations. In the worst-case scenario this will result in wrong conclusions.

Statistics that were developed for unconstrained data (the most prominent being correlation), can lead to spurious results when applied to relative data Pearson (1897). Interestingly, in geochemistry, where data often are percentages of chemical compounds in rock samples, this kind of problem was reported decades ago and eventually addressed using log-ratio analysis Aitchison (2003). Log-ratio analysis uses log-ratio transformations to take the data from the simplex to real space, thus avoiding many of the problems associated with constrained data. Genomic research is a prime target for such methods. Their non-reliance on semi-arbitrary normalization procedures makes it possible to bypass problems when comparing data produced across a wide-range of conditions. Such an approach has the potential to significantly broaden analysis prospects by allowing the systematic re-analysis and consolidation of existing data sets. It will be especially useful in situations where the large number of experimental conditions or the involvement of different laboratories The ENCODE Project Consortium (2011),Lonsdale (2013) make it virtually impossible to perform all experiments under the exact same conditions.

**Fig. 1:**
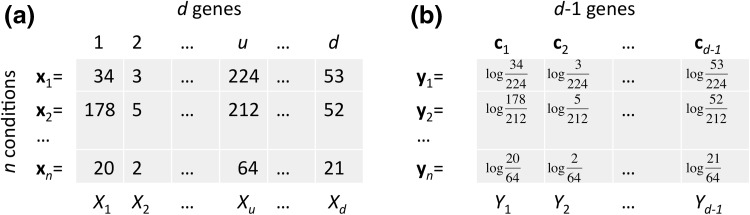
**a** Relative gene expression data matrix. Each row is considered a composition, the data can be formalized as sampled from a random composition consisting of random variables $X_j$. **b** Data matrix after alr transformation using the *u*-th column of the original matrix as a reference.

Let us now consider an $n\times d$ gene-expression data matrix where *d* genes correspond to the columns and the *n* (multivariate) observations are displayed in the rows. (Usually, this is a “fat” matrix in the sense that *d* is one or two orders of magnitude greater than *n*.) The observations are made under different experimental conditions or for a variety of genotypes, and for each such library, the expression values sum to a constant that is unrelated with the absolute amount of mRNA in the cells of origin. Each row in our matrix is thus considered a composition (to make each row a composition in the formal sense, we can divide all entries by the respective row sum, but this is unnecessary for the analysis proposed here). A row is denoted by a vector $\mathbf {x}$ whose elements $x_i$ are the gene expressions for genes $i=1,\ldots ,d$ in the given condition. Such data are usually counts of sequencing reads mapped to the genomic locations in question, and their precise nature is not of interest to us here. The only condition they have to fulfill is that ratios between values from the same condition are maintained from the original data (which is the case after multiplication of a normalization factor but not after applying a quantile normalization). Additionally, due to the need for applying logarithms, we may want to consider the application of pseudocounts to the expression values or, alternatively, restrict the analysis to submatrices that do not contain zeroes. To make our treatment sufficiently general, we will consider our matrix an *n*-sample of a *d*-part random composition $(X_1,\ldots ,X_d)$. In this setting, a gene corresponds to a (compositional) random variable. On the other hand, a gene *j* can be visualized as the column vector of its *n* observations $(x_{1j},\ldots ,x_{nj})^T$ (see Fig. 1 a).

To motivate our interest in proportionality, let us consider the underlying absolute data for a moment. Let $x_i=a_i/s$, with $a_i$ denoting the absolute mRNA amount from gene *i* in the given condition and *s* the total mRNA amount in this condition: $s={\sum\nolimits_{j=1}^{d}}a_j$. We neither know *s* nor the $a_i,$ but we have $x_i/x_j=a_i/a_j,$ so the only information maintained from the original absolute amounts is in the gene expression ratios. Gene pairs for which these ratios stay constant across conditions can thus be correctly inferred even on relative data. Taking the log of these ratios makes them symmetric with their reciprocal values. While correlations between the columns of our compositional matrix cannot be defined coherently, the covariance structure of a compositional data matrix can be summarized considering, for all pairs *i*, *j* ($i<j$), the (sample) variances of their log ratios log$\frac{x_i}{x_j}$ Aitchison (2003). These will be close to zero if genes *i* and *j* maintain an approximately proportional relationship $x_i\simeq m x_j$ across observations for some real value *m*.

In this contribution, we will interpret log-ratio transformations as an attempt to back transform relative data into absolute data. This point of view is not usually adopted, but it makes a connection with data normalization, a well-established field in genome research. In the first section of this paper, where we profit greatly from the treatment in Lovell et al. (2015), we show how proportionality can be measured as a kind of scaled log-ratio variance on absolute data. We then show that doing a log-ratio transformation with a very specific (“unchanged”) reference, on relative data we can detect all the proportional pairs defined on absolute data (“absolutely proportional set”) previously. This transformation, however, in fact is a normalization, and the information we need to perform it is usually not available. We show that small deviations from this unchanged reference will result in a small adjustment to the cut-off on our measure of proportionality to obtain a subset of the absolutely proportional set. The following section deals with the more common case of references that deviate greatly from the unchanged one. In this case it is hard to approximate the set of absolutely proportional pairs, and it is more difficult to avoid pairs that are called proportional although they were not proportional on the absolute data (“spurious” proportionality). We will give an exact result about the conditions under which prediction on relative data will coincide with the one on absolute data. In the following section, a slight generalization of proportionality leads to the concept of “partial” proportionality, a definition adopted from partial correlations. Finally, in the last section of the paper, we will apply the discussed concepts to a brief re-analysis of the data set used in Lovell et al. (2015). Here, in good agreement with our analytical results, the approach taken by Lovell et al. leads to a much lower overlap of prediction between absolute and relative data compared with the application of an approximately unchanged reference.

## Methods and results

### Scaling log-ratio variance on absolute data

In this section we show how we can identify a set of proportional gene pairs on (usually unavailable) absolute data. We will later investigate to what extent we can identify this set on the (available) relative data. Let us start with defining the “absolute” random variables $A_i={\text{log}}(sX_i),$ where *s* was defined in the introduction as the original absolute mRNA amount in a given condition. As each draw of the random variables $A_i\, (i=1\ldots d)$ corresponds to a condition, we now have to redefine *s* as a random variable $s:={\sum\nolimits_{i=1}^{d}}e^{A_i}$. Note also that we want to find proportionality between $sX_i$ and $sX_j$, but it is more convenient to work with the logs.

Unlike correlation, but similar to covariance, the log-ratio variance var($$A_i-A_j$$) has no intrinsic scale that makes its size intuitive to the analyst. Aitchison’s own proposal Aitchison (2003) to use $$1-\mathrm {exp}(-\sqrt{\mathrm {var(}A_i-A_j})$$ just achieves to limit the range to values between zero and one. With the stochasticity of gene expression data in mind, however, it is much more interesting to put log-ratio variance in relation to the size of the single variances involved: In the case of high variances, we are likely to consider higher values of var($$A_i-A_j)$$ still relevant and are inclined to apply a less stringent cut-off on it. This can be seen as the idea behind the scaling used in a recent work by Lovell et al. Lovell et al. (2015), where the following statistic is proposed:

1 $$\begin{aligned} \phi (A_i,A_j):=\frac{\mathrm {var}(A_i-A_j)}{\mathrm {var}(A_i)}=1+\beta ^2-2\beta ~r. \end{aligned}$$

**Fig. 2:**
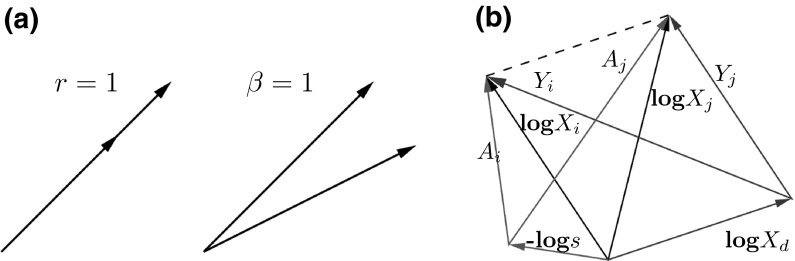
**a** Two vectors of observations when their goodness-of-fit parameters are $r=1$ (same direction) and $\beta =1$ (same length), respectively. Their log-ratio variance is the squared length of the link connecting them (here, roughly of the same size in both cases). **b** The relative data log$X_i$, log$X_j$ (*black vectors*) transformed to (logged) absolute data $A_i$, $A_j$ by the normalization −log*s* (*green vectors*) and log-ratio transformed using the reference $X_d$ to $Y_i$, $Y_j$ (*red vectors*). Note that the log ratio between $X_i$ and $X_j$ (*dashed line*) remains the same under both transformations (colour figure online)

Here, $\beta =\sqrt{\mathrm {var}(A_j)/\mathrm {var}(A_i)}$, and *r* is the correlation coefficient between $A_i$ and $A_j$. (Like Lovell et al. we drop the indices *i*, *j* from $\beta$ and *r*. For convenience, we reproduce their derivation in the "Appendix".) Interestingly, $\beta$ happens to be the absolute value^1^ of the estimated slope when plotting $A_i$ and $A_j$ against each other. This estimate of the slope is known as standardized major axis estimate Taskinen and Warton (2011). $\phi$ thus establishes a direct connection with line fitting for the scatter plot $A_i$ vs. $A_j$, where both $\beta$ and *r* have to be one for full proportionality (see Fig. 2a). Note that the centred *n*-sample vectors in our data matrix have a squared length corresponding to their variance. If logarithms are taken, log-ratio variances correspond to the squared lengths of difference vectors, which allows for intuitive representations such as the ones in Fig. 2.

A better alternative to $\phi$ is also mentioned in Lovell et al. but is not used there. It is defined by

2 $$\begin{aligned} \rho (A_i,A_j)=\frac{2~\mathrm {cov}(A_i,A_j)}{\mathrm {var}(A_i)+\mathrm {var}(A_j)}. \end{aligned}$$

This is a special case of a coefficient that was proposed in the context of determining reproducibility of measurements Lin (1989). The slightly more general “concordance correlation coefficient” also takes into account the shift due to the intercept, something irrelevant to detecting proportionality, as it only affects the size of the proportionality factor. The coefficient given in (2) can be understood as a scaling of the covariance that is similar to the one used when evaluating the correlation coefficient (with the geometric mean of the variances replaced by their arithmetic mean). The measure has the advantage of being symmetric in its arguments, of having a range from −1 to 1 (where −1 is attained for $sX_i$*reciprocal* to $sX_j$), and also of having a simpler relationship with $\beta$ and *r*. All in all, it seems a more generic measure that can be generalized more easily (as will be done in "Partial proportionality"). We collect some (straight-forward) identities involving this “proportionality coefficient” in the following

#### **Proposition 1**

(i) $$\rho (A_i,A_j) =1-\frac{\mathrm {var}\left( A_i-A_j\right) }{\mathrm {var}(A_i)+\mathrm {var}(A_j)}$$,
(ii) $$\rho (A_i,A_j) = \frac{2r}{\beta +1/\beta }$$,
(iii) $$\rho (A_i,A_j) = 1-\frac{2}{1+1/\tilde{\beta }^2}=\frac{1-\tilde{\beta }^2}{1+\tilde{\beta }^2}$$,
(iv) $$\rho (A_i+A_j,A_i-A_j) = \frac{1-\beta ^2}{1+\beta ^2}$$,

where for (iii) we defined $\tilde{\beta }=\sqrt{\mathrm {(var}(A_i-A_j)/\mathrm {(var}(A_i+A_j)}.$

(See "Appendix" for the proofs of all propositions.) The first statement gives a direct relationship with log-ratio variance and shows the similarity of $\rho$ with $\phi$ [more explicitly, we have $\phi =(1-\rho )(1+\beta ^2)].$ Given that var($A_i-A_j$) can reach a maximum of twice the sum of both variances (in the case of reciprocal $sX_i$, $sX_j$), it makes sense to use this sum for scaling it. The second of the identities above shows that we can obtain $\rho$ from the correlation coefficient by multiplying by a factor involving $\beta$. This factor is the geometric mean divided by the arithmetic mean of the variances, a function that attains one in case of equality of the variances and otherwise is smaller one. Statement (iii) shows that we can even express $\rho$ as a function of a single parameter $\tilde{\beta }$. This parameter equals $\beta$ in its functional form but depends on transformed variables that are the sum and the difference of the original ones. These transformed variables can be understood geometrically as the diagonals of the parallelogram spanned by the original vectors. Their variances can be understood in terms of a decomposition of total variance into group variance and within-group variance. Interestingly, their ratio is all we need, so the scaling of proportionality can be interpreted as relating within-group variance with group variance. Statement (iv) finally reveals an interesting duality involving the proportionality coefficients of the transformed variables and the original variables.

### Detecting proportional pairs on relative data using an unchanged reference

The problem with a scaling or a cut-off on log-ratio variance that depends on the individual variances is that these variances cannot be defined in a useful way on compositional data. Only variances of ratios can be defined coherently with respect to subcompositions, so we have to put $X_i$ and $X_j$ in relationship with a third variable or a term involving other variables. A first step in compositional data analysis is thus to do a log-ratio transformation of the data matrix and by this removing its constant-sum constraint. For this, a number of options are available. Perhaps the most simple and intuitive is to divide each entry by one of the components (which is thereby chosen as the reference). This additive log-ratio transformation is applied to each row in our data matrix by

3 $$\begin{aligned} \mathrm {alr}(\mathbf {x})=\left( \mathrm {log}(x_1/x_d),\dots ,\mathrm {log}(x_{d-1}/x_d)\right) . \end{aligned}$$

It results in vectors with dimension reduced by one compared with the original rows, so the reference is “sacrificed”. The *j*-th column of our alr-transformed data matrix will now be

4 $$\begin{aligned} \mathbf {c}_j=\left( \mathrm {log}(x_{1j}/x_{1d}),\dots ,\mathrm {log}(x_{nj}/x_{nd})\right) ^T. \end{aligned}$$

The column vectors $\mathbf {c}_j$ we can consider an *n*-sample of (transformed) scalar random variables $Y_j$ (see Fig. 1b). While log-ratio variances ${\text{var}}(Y_i-Y_j)={\text{var(log}}(X_i/X_j))$ remain unaffected by the transformation, individual variances ${\text{var}}(Y_i)={\text{var(log}}(X_i/X_d))$ are log-ratio variances themselves and thus depend on the choice of the reference.

Given a set of all proportional gene pairs inferred on the absolute data (for a given cut-off on *ρ*), are we able to detect them on the compositional data applying the coefficient discussed in the previous section? For a resounding yes, we would need a very specific reference that effectively back transforms the data to absolute data (see Fig. 2b). To see this, let us spell out the terms that contribute to the transformed compositional data:

5 $$\begin{aligned} Y_i=\mathrm {log}X_i-\mathrm {log}X_d=A_i-\mathrm {log~}s-\mathrm {log}X_d=A_i-\mathrm {log~}sX_d=A_i-A_d. \end{aligned}$$

If $sX_d$ is constant, we recover the results from the absolute data. Genes $X_d$ fulfilling this would have a variance just reproducing the shift *s* needed to normalize the data, i.e. they would be unchanged across conditions in the absolute data (i.e. var$(A_d)=0).$ (Note that $A_d$ in Fig. 2b is given by the link between the vectors corresponding to−log*s* and log$X_d$, pointing towards the latter.) Such a reference could be a housekeeping gene that is known to be unchanged under the conditions considered. This is of course an idealization, and we need to know what happens when this reference gene reproduces the shift up to a small error. The following expansion links the proportionality coefficient obtained on the absolute data with the one after transformation of the relative data using such an approximately unchanged reference:

#### **Proposition 2**

*Let*$A_i=\mathrm {log}~sX_i$* be the original absolute amounts of the alr-transformed variables*$Y_i=\mathrm {log}(X_i/X_d),$* where for the reference we have*$\mathrm {log}X_d=-\mathrm {log} s+\epsilon.$* Then with*$\varphi (\epsilon )=\sqrt{\mathrm {var}(2\epsilon )/\left( \mathrm {var}(A_i)+\mathrm {var}(A_j)\right) }$*we have*

$$\begin{aligned} \rho (Y_i,Y_j)=\rho (A_i,A_j)-\mathrm {corr}(A_i+A_j,\epsilon )\sqrt{\left( 1-\rho ^2(A_i,A_j)\right) \left( 1-\rho (A_i,A_j)\right) }\varphi (\epsilon )\\ -(1-\rho (A_i,A_j))\left( \mathrm {corr}^2(A_i+A_j,\epsilon )\left( 1+\rho (A_i,A_j)\right) -\frac{1}{2}\right) \varphi ^2(\epsilon )+O(\varphi ^3(\epsilon )). \end{aligned}$$

As can be seen from the expansion, the direction of the reference with respect to the pair (as given by the correlation coefficient) will decide if the proportionality increases or decreases. Without this information, the coefficients of the expansion can still be easily bounded. They become increasingly small for $\rho (A_i,A_j)$ close to 1. Considering pairs with a cut-off $\rho (A_i,A_j)\ge 0.98$ proportional, by how much do we have to raise the cut-off on the relative data to avoid all pairs with $\rho (A_i,A_j)<0.98$ but $\rho (Y_i,Y_j)\ge 0.98$ (i.e. false positives)? We have approximately

6 $$\begin{aligned} \rho (Y_i,Y_j)\le 0.98+\varphi (\epsilon )/35. \end{aligned}$$

For easier interpretation, let us introduce a parameter *C* defined as the ratio of the average variance of the gene pair with the variance of our reference:

7 $$\begin{aligned} C:=\frac{\mathrm {var}(A_i)+\mathrm {var}(A_j)}{2\mathrm {var}(\epsilon )}. \end{aligned}$$

Using $C=1/(2\varphi ^2(\epsilon ))$, we find that

8 $$\begin{aligned} \rho (Y_i,Y_j)\le 0.98+\frac{1}{35\sqrt{2C}}. \end{aligned}$$

**Fig. 3:**
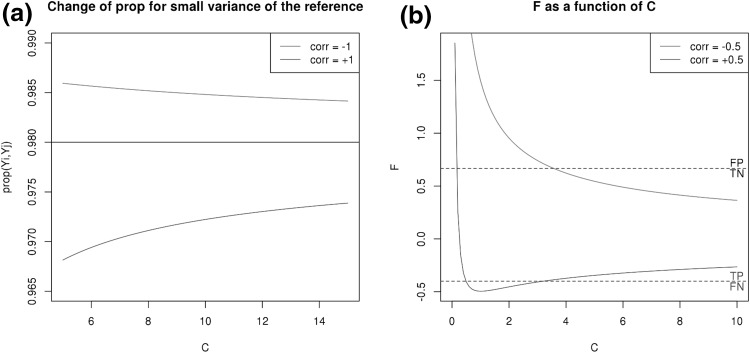
**a** Proportionality coefficient on relative data as a function of *C* according to the expansion of Proposition 2, for $\rho (A_i,A_j)=0.98$ and for two different correlation coefficents with the reference. **b** Illustration of Corollary 2. $F(A_d)$ as a function of *C* is shown for $\rho (A_i,A_j)=0.98$ and two different correlation coefficents with the reference. Two cut-offs ($K=0.975$ and $K=0.985$) result in $\tilde{K}$ (dashed lines) separating different regimes of accuracy for $\rho (Y_i,Y_j)$ (colour figure online)

Increasing the cut-off to 0.99, we would still avoid pairs that have a $\rho (A_i,A_j)$ of almost 0.98 and whose members have an average variance at least $C=5$ times higher than the reference (see Fig. 3a). This is usually sufficient, as pairs with variances close to $\epsilon$ do not achieve high proportionality coefficients (see also Fig. 7a in the last section) and can be considered to belong to the set of unchanged genes. Note however that this is an expansion in $\varphi$, not in $\epsilon$, so strictly speaking it applies only if *C* is sufficiently big (we will give an exact result for all *C* in the next section). Qualitatively, we can see already that taking a sufficiently high cut-off should lead to a set of pairs that were also proportional on the absolute data. A similar argument applies for false negatives (where the required adjustment should be small, as pairs that are above the cut-off on relative data in practice have higher *C*).

Lovell et al. Lovell et al. (2015) propose to use the mean over the log $X_i$ as a reference. This is known as the centred log-ratio transformation:

9 $$\begin{aligned} \mathrm {clr}(\mathbf {x})=\left( \mathrm {log}(x_1/g(\mathbf {x})),\dots ,\mathrm {log}(x_d/g(\mathbf {x}))\right) , \end{aligned}$$

with $g(\mathbf {x})$ the geometric mean over the genes for the given condition. The problem with this transformation is that it is sub-compositionally incoherent Aitchison (2003), so results will change to some extent when using subsets of genes for the analysis. In some cases, $g(\mathbf {x})$ can approximate an unchanged reference. This applies whenever the majority of genes remains unchanged across conditions, so the unchanged genes will dominate the behaviour of the reference. Note that this is also the condition needed for a normalization by effective library size Robinson and Oshlack (2012).

### Measuring proportionality on relative data using a changing reference

What if the reference itself is changing on the absolute data? Depending on (size and direction of) the variance of the reference, only a part of the pairs that are proportional on the absolute data can now be identified, and a certain number of pairs that were not proportional on the absolute data will be declared proportional on the relative data. More precisely, we have the following

#### **Proposition 3**

*Let*$A_i=\mathrm {log}~sX_i$* be the original absolute amounts of the alr-transformed variables*$Y_i=\mathrm {log}(X_i/X_d).$* We then have*

10 $$\begin{aligned} \rho (Y_i,Y_j)-\rho (A_i,A_j)=\frac{1-\rho (A_i,A_j)}{1+2/F(A_d)}, \end{aligned}$$

*where*$$F(A_d)=\varphi ^2(A_d)-2\sqrt{1+\rho (A_i,A_j)}\mathrm {corr}(A_i+A_j,A_d)\cdot \varphi (A_d)$$*and*$$\varphi (A_d)=\sqrt{\mathrm {var}(2A_d)/\left( \mathrm {var}(A_i)+\mathrm {var}(A_j)\right)}.$$

If $\rho (Y_i,Y_j)$ increases with respect to $\rho (A_i,A_j)$, we maintain pairs that were absolutely proportional, but we also introduce false positives.

#### **Corollary 1**

$$\rho (Y_i,Y_j)\ge \rho (A_i,A_j)$$*iff*$$\mathrm {corr}(A_i+A_j,A_d)\le \sqrt{\frac{\mathrm {var}(A_d)}{\mathrm {var}(A_i+A_j)}}.$$

We see that $\rho (Y_i,Y_j)$ increases for negative correlations with the reference, as well as for references having a variance that exceeds the group variance $\mathrm {var}((A_i+A_j)/2)$ of the pair by a factor 4.

#### **Corollary 2**

*Let the set of true positives*$\mathcal {S}_{\mathrm {TP}},$*false positives*$\mathcal {S}_{\mathrm {FP}},$*true negatives*$\mathcal {S}_{\mathrm {TN}}$*and false negatives*$\mathcal {S}_{\mathrm {FN}}$*for a given cut-offK*$(1>K>0)$*on the proportionality coefficient be defined by*

- $$\mathcal {S}_{\mathrm {TP}}=\left\{ (i,j):\rho (A_i,A_j)\ge K, \rho (Y_i,Y_j)\ge K\right\}$$,
- $$\mathcal {S}_{\mathrm {FP}}=\left\{ (i,j):\rho (A_i,A_j)< K\le \rho (Y_i,Y_j)\right\}$$,
- $$\mathcal {S}_{\mathrm {TN}}=\left\{ (i,j):\rho (A_i,A_j)< K, \rho (Y_i,Y_j)< K\right\}$$,
- $$\mathcal {S}_{\mathrm {FN}}=\left\{ (i,j):\rho (Y_i,Y_j)< K\le \rho (A_i,A_j)\right\}$$. Also, let
- $\tilde{K}:=2(K-\rho (A_i,A_j))/(1-K)$. *Then for a given pair of genes i, j we have*

(i) $$(i,j)\in \mathcal {S}_{\mathrm {TP}}\Longleftrightarrow F(A_d)\ge \tilde{K}\le 0$$,
(ii) $$(i,j)\in \mathcal {S}_{\mathrm {FP}}\Longleftrightarrow F(A_d)\ge \tilde{K}>0$$,
(iii) $$(i,j)\in \mathcal {S}_{\mathrm {TN}}\Longleftrightarrow F(A_d)< \tilde{K}>0,$$
(iv) $$(i,j)\in \mathcal {S}_{\mathrm {FN}}\Longleftrightarrow F(A_d)< \tilde{K}\le 0.$$

To determine the fate of a gene pair analytically, we thus have to solve the quadratic equation for $F(A_d)-\tilde{K}.$ For easier interpretation, we can again replace $\varphi$ by the parameter $C=\left( \mathrm {var}(A_i)+\mathrm {var}(A_j)\right) /2\mathrm {var}(A_d)$ as we did in (7). In Fig. 3b it is shown, for a gene pair with $\rho (A_i,A_j)=0.98,$ how *F* changes with *C* in case of positive and negative correlation with the reference. Two boundaries $\tilde{K}$ are shown for cutoffs $K=0.975$ and $K=0.985,$ and the resulting regimes of FP, TN, TP and FN are indicated. An important subset of false positive pairs are the ones from the set of unchanged genes:

#### **Corollary 3**

*Let*$\epsilon$*be an error term characterizing the stochasticity of unchanged genes, i.e. for such genesiwe have*$\mathrm {var}(A_i)\le \mathrm {var}(\epsilon).$*LetK*$(0<K<1)$*be the cut-off on*$\rho$*above which genes are proportional on the absolute data. Then pairs of unchanged genes with*$\rho (A_i,A_j)<K$*will be proportional on the relative data, i.e.*$\rho (Y_i,Y_j)\ge K$*for a reference fulfilling*

$$\begin{aligned} \mathrm {var}(A_d)\ge \frac{8\mathrm {var}(\epsilon )}{1-K}.\nonumber \end{aligned}$$

Finally, we can again ask if there is a sufficiently high cut-off on $\rho (Y_i,Y_j)$ to avoid all false positives. Choosing as an example again $\rho (A_i,A_j)=0.98$, we get an upper bound on $F(A_d)$ by

11 $$\begin{aligned} F(A_d)\le \frac{1}{2C}+\frac{2}{\sqrt{C}}. \end{aligned}$$

In the worst case, the gene pair has a low variance almost of the order of the unchanged set but reaches a $\rho (A_i,A_j)$ close to 0.98. Let us assume our reference has a variance that is five times higher than the average variance of such a pair. With $C=1/5$ (i.e. $F\le 7$) we get

12 $$\begin{aligned} \rho (Y_i,Y_j)\le 0.98+\frac{0.02}{1+2/7}\le 0.996. \end{aligned}$$

So even if the reference has a relatively low variance, the cut-off will get quite close to 1 when trying to avoid pairs that are not proportional on the absolute data. The situation can be much worse for false negatives: The function $\rho (Y_i,Y_j)-\rho (A_i,A_j)$ is bounded below by $-(1+\rho (A_i,A_j))$ at $F=-(1+\rho (A_i,A_j))$, which in turn is attained for $\varphi (A_d)=\sqrt{1+\rho }$ or $C=1/(2(1+\rho (A_i,A_j))$ (c.f. Lemma 1 in the "Appendix"). In our example, *C* is pretty close to this minimum, and a lower bound on $\rho (Y_i,Y_j)$ reaches 0.2, a cut-off that would give a sensitivity of 100 % but would be useless in terms of specificity.

Although raising the cut-off will generally lead to sets of pairs that are more likely to be proportional on the absolute data, we usually do not have the information to know what is a good cut-off (and it might be so high that no pairs remain). We saw that a sufficiently high absolute variance of our reference introduces proportional pairs that are spurious (in the sense that on the absolute data they are not proportional). However, usually we do not have information about the size of the variance of the reference (on the absolute data). We ended up in a situation similar to the one described in the classical work of Pearson Pearson (1897), where it is shown how spurious correlation is introduced between two variables due to the common division by a third variable^2^. Given that log-ratio analysis sets out to solve exactly the problem of spurious correlation, the fact that scaling the log-ratio variance re-introduces a similar problem (spurious proportionality) appears rather unsatisfactory.

### Partial proportionality

In the previous sections, we were using the fact that the size of the log-ratios (their variance) is identical on absolute and relative data, and we investigated the effect of a scaling. Also the direction of the log-ratios is identical on both types of data. Correlation coefficients between log-ratios are thus identical between absolute and relative data, and log-ratio scatter plots are one of the available tools when analyzing compositional data sets Greenacre (2010),van den Boogart and Tolosana-Delgado (2013). In this section, we will show that such correlations can be interpreted in terms of a slight generalization of proportionality.

**Fig. 4:**
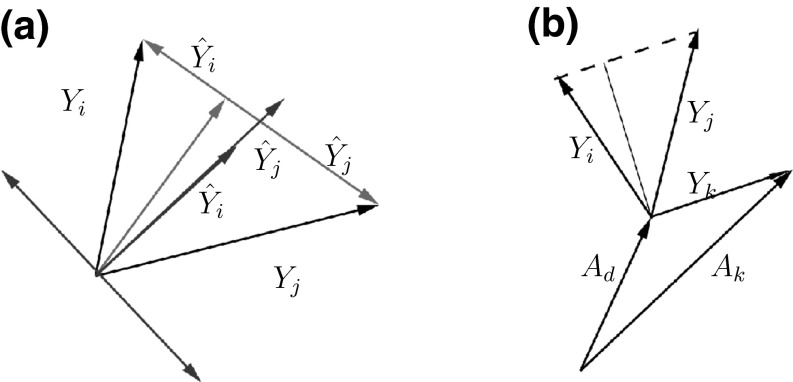
**a** Direction of the vector corresponding to $Y_k$ when it lies in the same plane as $Y_i$ and $Y_j$ and the partial proportionality coefficient is one (*green*) and minus one (*red*), repectively. If $Y_k$ is parallel to the difference (log ratio) between $Y_i$ and $Y_j,$ the linear least squares predictors $\hat{Y}_i$ and $\hat{Y}_j$ point in opposite directions (*green labeled vectors*), and the residuals $Y_i-\hat{Y}_i,$ $Y_j-\hat{Y}_j$ coincide in the *green, unlabeled vector*. If $Y_k$ is parallel to the sum of $Y_i$ and $Y_j$, the linear least squares predictors $\hat{Y}_i$ and $\hat{Y}_j$ point in the same direction (*red labeled vectors*), and the residuals $Y_i-\hat{Y}_i$, $Y_j-\hat{Y}_j$ have the same length and point in opposite directions (*unlabeled red vectors*). **b** Log ratios between $X_i$ and $X_j$ and between $X_k$ and $X_d$ are parallel whenever $Y_i$ and $Y_j$ are partially proportional wrt. $Y_k$ (colour figure online)

The simple functional relationship with the correlation coefficient given in Proposition 1 (ii) suggests a straight-forward extension of our definition along the lines of a related measure, namely partial correlation. Partial correlations have been used extensively in the construction of gene networks because, in theory, they allow for identification of direct pairwise interactions that are not mediated by other genes, and techniques have been developed for inverting the (regularized) correlation matrix Schäfer and Strimmer (2005) to obtain them. Let us restrict the problem to partialling on a single gene *k* here. Geometrically speaking, for our transformed data matrix, a partial correlation between genes *i* and *j* wrt. gene *k* is obtained from projecting the vectors $\mathbf {c}_i$ and $\mathbf {c}_j$ onto the plane perpendicular to the vector $\mathbf {c}_k$. In these projections, the part of the correlation between $\mathbf {c}_i$ and $\mathbf {c}_j$ that was due to the correlation with $\mathbf {c}_k$ is removed. In more general terms of multiple regression, projecting along $Y_k$ yields the linear least squares predictors $\hat{Y}_i$ and $\hat{Y}_j$ wrt. $Y_k,$ and partial correlations are obtained from correlating the projections onto the orthogonal plane, i.e. correlating the residuals $Y_i-\hat{Y}_i$ and $Y_j-\hat{Y}_j.$ Note that if we talk about projections of scalar random variables, we have the *n*-sample vectors in mind. This is also the way in which Fig. 4 should be understood.

What is the relationship of partial correlation with proportionality? Replacing the correlation coefficient by the partial correlation coefficient in Proposition 1 (ii), and adjusting $\beta$ accordingly, we obtain a natural definition for a partial proportionality coefficient.

More precisely, we have the following equivalents to the first three identities of Proposition 1:

#### **Proposition 4**

*Let*$Y_i, Y_j, and Y_k$*be scalar random variables, and let*$\hat{Y}_i$*and*$\hat{Y}_j$*be the linear least-squares predictors of*$Y_i$*and*$Y_j$*wrt.*$Y_k.$*Then*

(i) $$\rho \left( Y_i-\hat{Y}_i,Y_j-\hat{Y}_j\right) = 1-\frac{\mathrm {var}(Y_i-Y_j)\left( 1-\mathrm {corr}^2(Y_i-Y_j,Y_k)\right) }{\mathrm {var}(Y_i)\left( 1-\mathrm {corr}^2(Y_i,Y_k)\right) +\mathrm {var}(Y_j)\left( 1-\mathrm {corr}^2(Y_j,Y_k)\right) }.$$
(ii) $$\rho \left( Y_i-\hat{Y}_i,Y_j-\hat{Y}_j\right) =\frac{2(r-r_{ik}r_{jk})}{(1-r^2_{jk})\beta +(1-r^2_{ik})/\beta }$$
(iii) $$\rho \left( Y_i-\hat{Y}_i,Y_j-\hat{Y}_j\right) = 1-\frac{2}{1+G/\tilde{\beta }^2}=\frac{1-\tilde{\beta }^2/G}{1+\tilde{\beta }^2/G}$$

*where*$$\beta =\sqrt{\mathrm {var}(Y_j)/\mathrm {var}(Y_i)},$$$$r=\mathrm {corr}(Y_i,Y_j),$$$$r_{ik}=\mathrm {corr}(Y_i,Y_k),$$$$\tilde{\beta }=\sqrt{\mathrm {(var}(Y_i-Y_j)/\mathrm {(var}(Y_i+Y_j)},$$*and*$$G=\left( 1-\mathrm {corr}^2(Y_i+Y_j,Y_k)\right) /\left( 1-\mathrm {corr}^2(Y_i-Y_j,Y_k)\right).$$

(The proof follows immediately from some well-known identities involving variances of the least squares predictor, see "Appendix".) Clearly, if the direction we are partialling on is perpendicular to the plane of the gene pair, the partial coefficient coincides with the proportionality coefficient. The interesting cases occur for directions within the plane of the gene pair. From (i) it follows immediately that we have partial proportionality between *i* and *j* if $Y_k$ falls parallel to their log ratio (see the green vectors in Fig. 4a). From (ii) it follows that the partial coefficient will vanish if $Y_k$ is parallel with either $Y_i$ or $Y_j$. From (iii) it follows that we have partial reciprocality between *i* and *j* if $Y_k$ falls parallel to the sum of $Y_i$ and $Y_j$ (red vectors in Fig. 4a). However, only the first of the described cases has a simple relationship with the absolute data: Partial proportionality between *i* and *j* wrt. *k* when their common reference is *d* implies that the log ratio between $A_i$ and $A_j$ is parallel to the one between $A_k$ and $A_d$ (see Fig. 4b).

## Data analysis

**Fig. 5:**
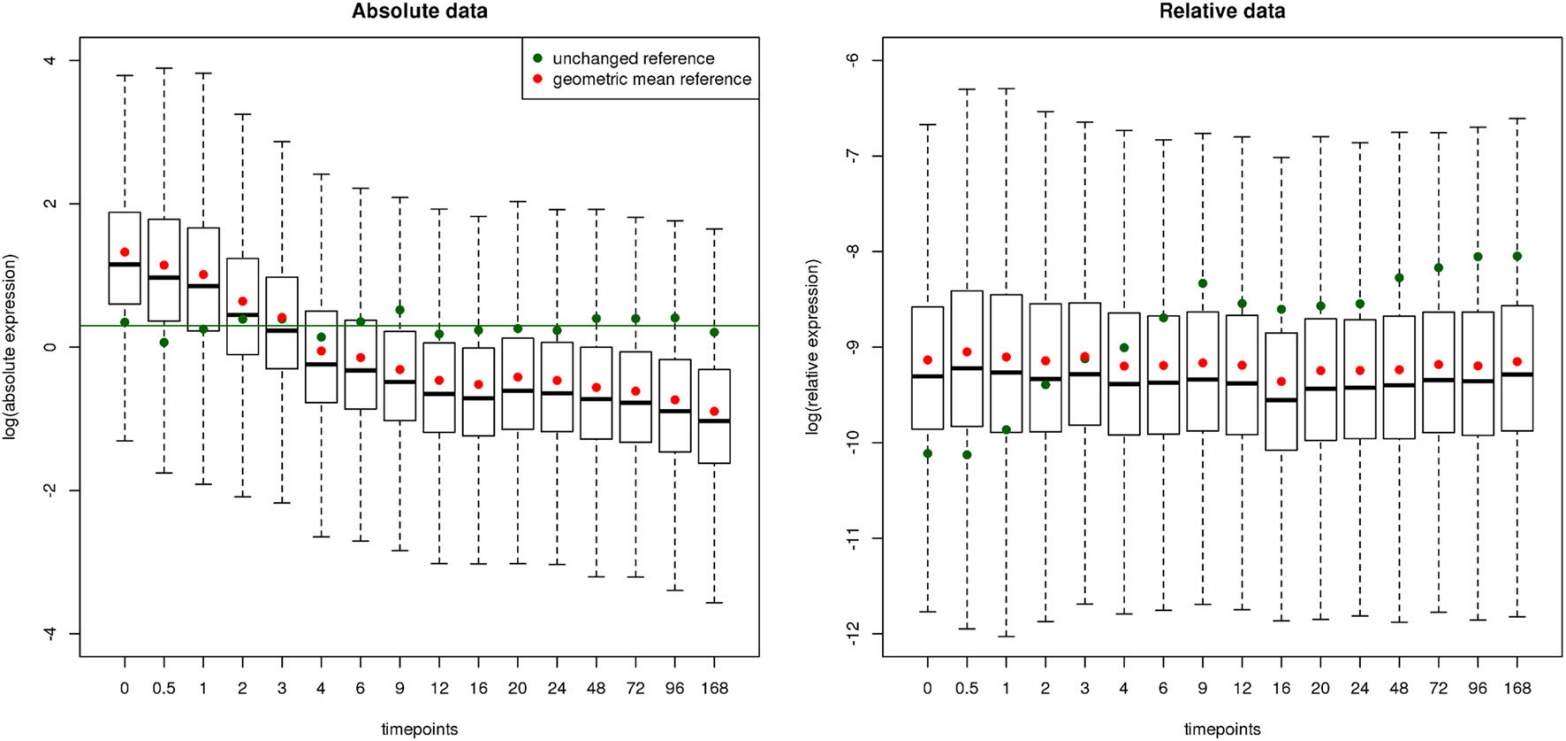
Absolute and relative gene expression data box plots. Each box summarizes the distribution of logged gene expressions in a given condition (time point) and, in the case of the relative data, corresponds to one row of the data matrix. *Left panel*: Overall absolute expression is going down with time, with (approximately) unchanged and geometric mean references behaving accordingly on the absolute data (the *green horizontal line* denotes the mean log expression of the unchanged gene). *Right panel* On the relative data, the unchanged reference appears to be going up, while the geometric mean reference appears to remain unchanged. To recover the absolute data, each box has to be shifted by an amount approximated by the distance of the *green dots* from the value indicated by the *green horizontal line* in the *left panel* (colour figure online)

In this section we will put into practice our theoretical considerations of the previous sections. We will use the data set provided in Marguerat et al. (2012) and re-analyzed by Lovell et al. Lovell et al. (2015), where we profit from the excellent documentation including the R-code made available in the latter. The data are from fission yeast cells entering quiescence after time point zero. The data at time point zero are counts obtained by RNA-seq that are supposed to be roughly proportional to the original absolute mRNA amounts. The data from the 15 subsequent time points are abundances relative to time point zero and were obtained by microarray. For biological and technical details we refer the reader to the original publication. The data set is not typical in the sense that we have both the relative data and an approximation of the absolute data at our disposal (Fig. 5). It is thus well suited to validate our theoretical considerations, especially regarding the overlap of predicted proportionality on absolute and relative data obtained by alternative analysis approaches. Note that the approach we propose differs from the one in Lovell et al. in two respects: in the use of the proportionality coefficient instead of the $\phi$ statistic, and in the application of an alternative log-ratio transformation. While the coefficient has some clear advantages over $\phi$ in that it is symmetric, has a limited range, can also detect reciprocality and allows for the definition of a partial coefficient, we will see that the main difference in outcome of our approach comes from the proposed log-ratio transformation.

**Fig. 6:**
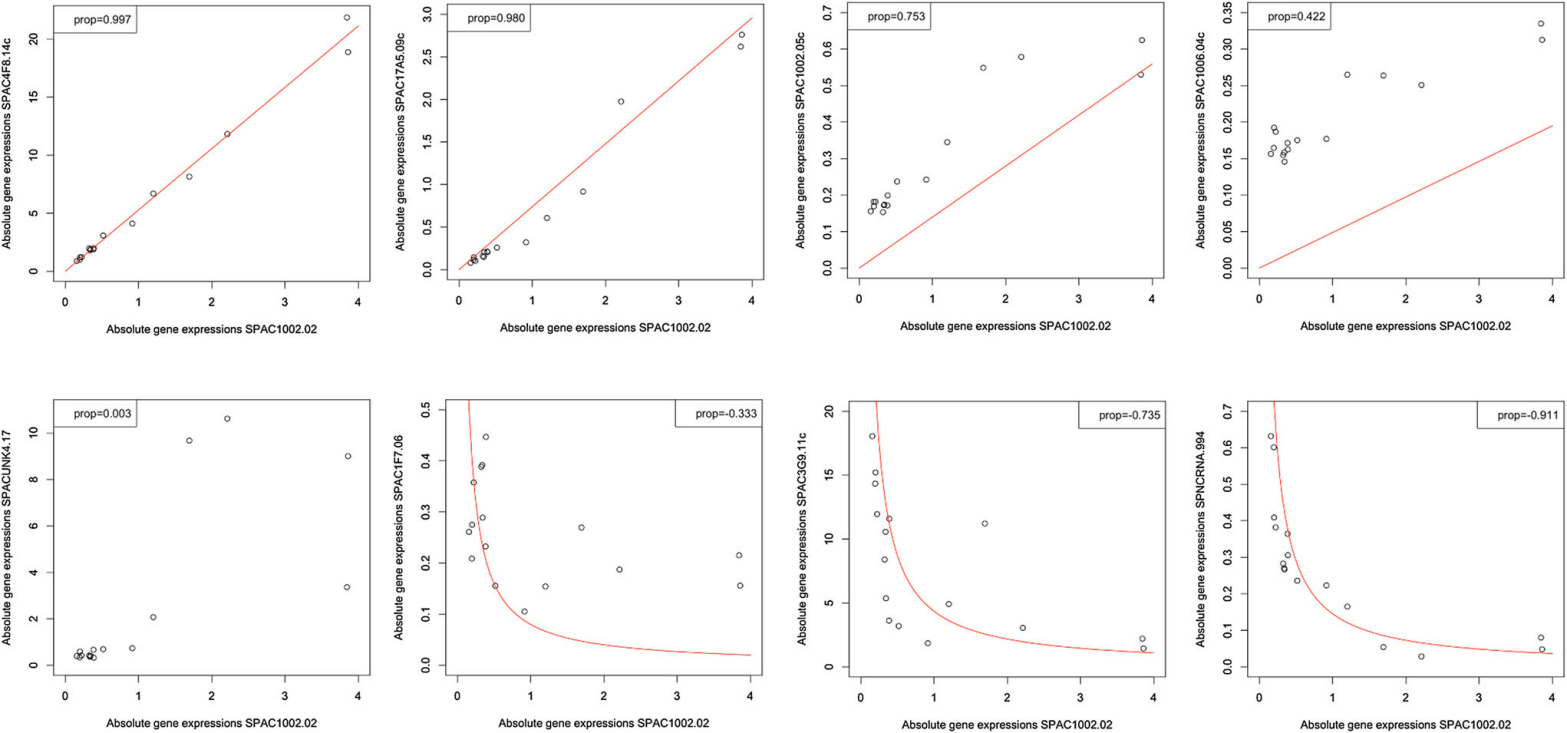
Absolute gene expression scatter plots of the gene SPAC1002.02 with various other genes and the corresponding proportionality coefficients. The *red lines* are fits of the slope (through the origin, corresponding to $\beta =1$), where the slope is estimated by $\sqrt{\mathrm {var(exp}(A_j))/\mathrm {var(exp}(A_i))}$. In the case of negative coefficients, this number is divided by exp$(A_i)$ instead of being multiplied by exp$(A_i)$. Note that the goodness of fit measures $\beta$ and *r* determining the size of the coefficient are for the logged data, which is not shown here (colour figure online)

As log-ratio transformations need non-zero entries throughout the data matrix, we follow Lovell et al. in using a subset of 3031 genes fulfilling this condition in all 16 time points. We start with studying the goodness of fit with the expected behaviour for proportional (and reciprocal) genes on the absolute data. Scatter plots for a variety of values of $\rho (A_i,A_j)$ (all for the same $A_i$) are shown in Fig. 6. As proportionality between genes is a property of the original data (where no logarithm is taken), we are not showing logarithmic scatter plots here, although the “goodness of fit” measures *r* and $\beta$ are used for fitting logarithms. To nevertheless give an impression of the quality of the fit, red lines with a slope corresponding to the variance ratio between the genes are drawn through the origin. This way both effects, the shift with respect to a zero intercept and how well the expected slope is reproduced can be studied independently. (For negative coefficients we divide the slope by the values of the gene on the horizontal axis instead of multiplying them.) We conclude that for absolute values of the coefficient increasingly close to one, the data seem to reproduce better and better the desired behaviour outlined by the red curves.

**Fig. 7:**
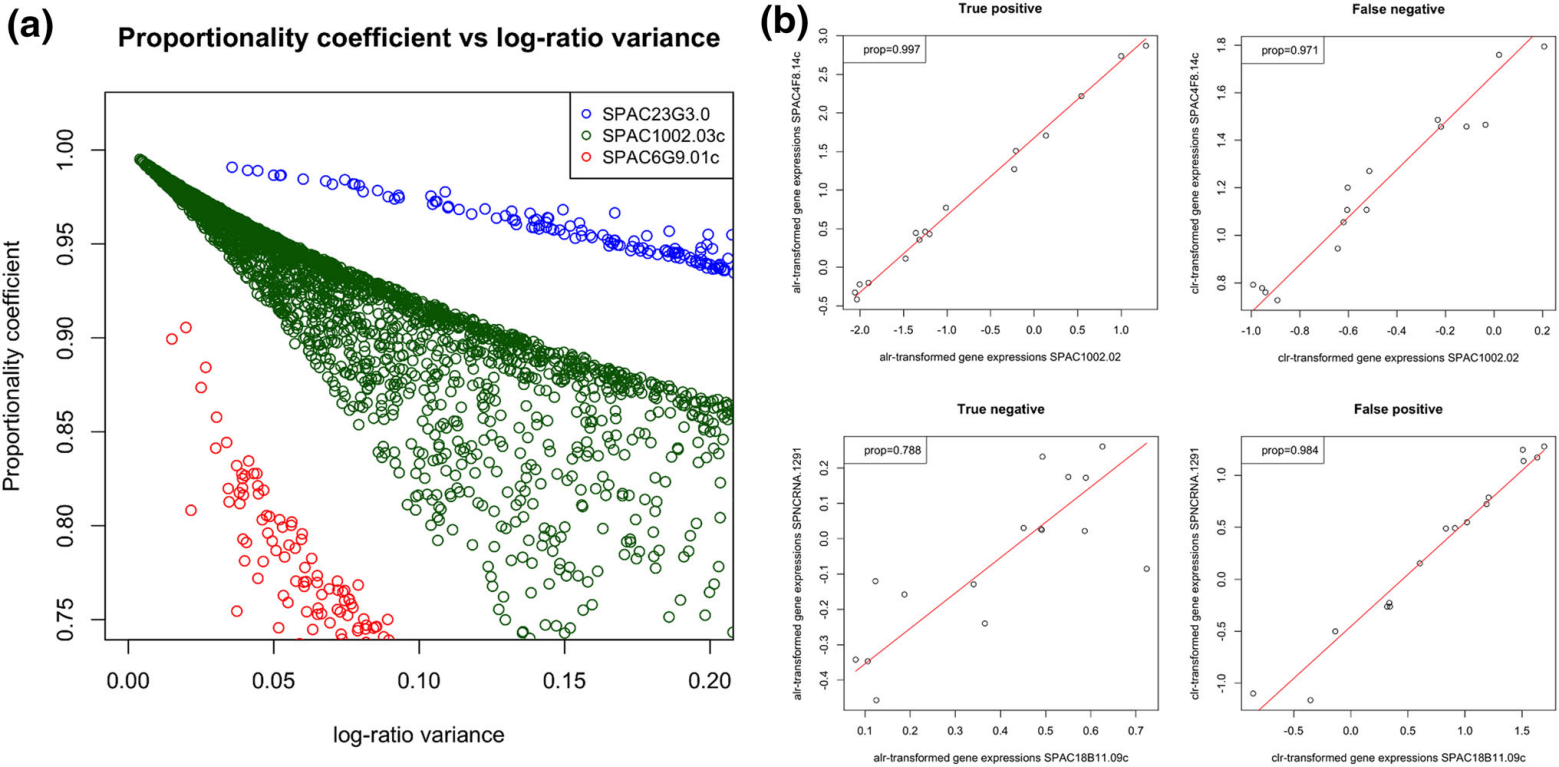
**a** Scaling of log-ratio variance using the proportionality coefficient. Shown are coefficients of gene pairs involving three genes with different variances versus their log-ratio variances (absolute data). The three genes have variances of the logged absolute data of 2.1 (*blue*), 0.39 (*green*) and 0.076 (*red*), respectively. Gene pairs with higher variance can have higher log-ratio variance to attain the same value of the coefficient. **b** Log-ratio (alr and clr) transformed gene expression scatter plots. The *red lines* are fits with a slope $\beta =1$ (the intercept is estimated by the mean over $Y_j-Y_i$). *Upper panels* A gene pair correctly identified as proportional (*left*) and incorrectly discarded (*right*). The same pair is shown in the first panel of Fig. 6. *Lower panels* A gene pair correctly discarded (*left*) and incorrectly identified as proportional (*right*) (colour figure online)

We next study the effect of the scaling of log ratios which the proportionality coefficient should achieve. In Fig. 7a, we show a scatter of proportionality coefficient vs. log ratio for gene pairs all containing one of three genes with different variances. It can be seen clearly that the gene with highest variance achieves higher values of the proportionality coefficient for the same log ratios. In fact, pairs involving the gene with the lowest variance do not make the cut-off of 0.98, although they reach log ratios that are closer to zero than those of pairs involving the high-variance gene. In practice, high cut-offs should make sure that pairs of unchanged genes achieving small log-ratios by chance due to their random fluctuations will not be called proportional. As desired, the gene with intermediate variance shows an intermediate slope. It attains, however, the smallest log ratios and highest coefficients due to its great number of partners that have a similar variance.

We then set out to study the relative data, which is truly compositional in the sense that it is normalized to one following Lovell et al. (2015). To do an alr transformation of this relative data matrix, we looked for a good candidate for an unchanged reference. We inspected the genes with lowest coefficient of variation *on the absolute data* by eye and chose the one with lowest bias towards a particular condition (green dots in Fig. 5). Interestingly, although the resulting gene Rev7 (SPBC12D12.09) can possibly be considered housekeeping (it is a subunit of DNA polymerase zeta), it is none of the usual candidates of unchanged genes (like Act1, Srb4, Rip1, Wbp1 used in the Affymetrix Yeast Genome Array 2.0), which are all changing greatly on this data set. The data is special in the sense that the set of unchanged genes is extremely small. This gives us, however, the opportunity to compare our reference with the geometric mean reference used by the clr transformation. The latter rather reflects the general downward trend observed by the majority of genes (see the red dots in Fig. 5).

The unchanged reference has a variance var($$A_d)=0.014$$ on the (logged) absolute data. Its variance on the relative data corresponds roughly to the shift log(*s*) due to the normalization, its value is var(log$$X_d))=0.49$$ (while the true var(log($$s))=0.45$$). Clearly, the situation is rather the opposite for the geometric mean reference: its variance on the relative data var(log$$(g))=0.005$$, while on the absolute data it achieves a variance of 0.51. Once the two log-ratio transformations are obtained, we can study again scatter plots corresponding to certain values of the proportionality coefficient, this time on the transformed data and with the red lines depicting the ideal slope of $$\beta =1$$. In the upper two panels of Fig. 7 b , data for a gene pair under the two transformations is shown (it is the same pair as in the upper left panel of Fig. 6). While under the alr transformation the value of the coefficient remains almost unchanged, it drops to 0.97 under the clr transformation. Taking the cut-off at 0.98, this results in a false negative. Another situation is shown in the lower panels. While the gene pair shown has a proportionality coefficient of 0.82 on the absolute data, under the alr transformation it slightly drops to a value of 0.79, while under the clr transformation it goes up to 0.98, thus resulting in a false positive.

**Fig. 8:**
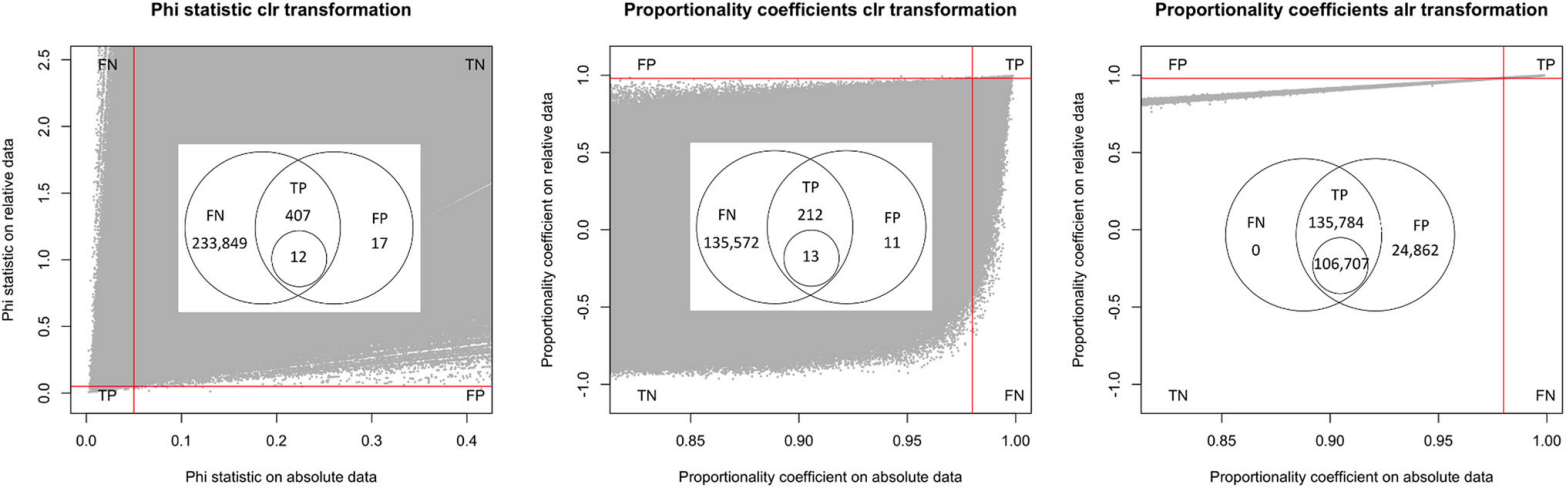
Prediction accuracy for the phi statistic (*left*), the proportionality coefficient using a clr transformation (*centre*) and the proportionality coefficient using the alr transformation with an unchanged gene (*right*). Each *grey dot* represents a gene pair. The applied cut-offs of 0.05 (for $\phi$) and 0.98 (for $\rho$) are denoted by *red vertical* and *horizontal lines*, and the resulting quadrants of false positives (FP), false negatives (FN), true positives (TP) and true negatives (TN) are indicated. The range was chosen so as to include all false positives. The insets in the centre are Venn diagrams, where the *left* and *right circles* denote the set of pairs found on absolute data and on relative data, respectively. Shown are the numbers of FN, TP and FP. The *circle* in the intersection denotes the largest set of pairs having 100 % specificity that can be obtained by just adjusting the cut-off. Cut-offs for these sets are <0.012, >0.994, and >0.983, respectively (colour figure online)

To obtain a general idea about the accuracy of our conclusions on relative data regarding proportionality on absolute data, we plotted values on transformed data versus values on absolute data for three measures: the proportionality coefficient under the two transformations as well as the statistic $\phi$ under the clr transformation considered by Lovell et al. (see Fig. 8). Venn diagrams in the insets of all panels show the precise numbers of FP, TP, and FN. The breadth of the scatters under clr transformations indicates the problems we face for references of high (absolute) variance and is in good agreement with our theoretical conclusions in the previous sections. While specificity for the clr transformation is rather good for the cut-offs considered (positive predictive value (PPV) is 96 % for $\phi$ and 95 % for $\rho$), the sensitivity (0.17 % for $\phi$ and 0.16 % for $\rho$) is very low. The situation is quite different under the alr transformation: The scatter shows a tight correlation. A PPV of 85 % is obtained at 100 % sensitivity. As we showed analytically, the absence of false negatives could be caused by a negative correlation of our reference gene with all proportional gene pairs. The sub-optimal PPV can be raised to 100 % by increasing the cut-off slightly (to 0.983) while loosing sensitivity (which goes down to 79 %). Of course, the comparison with the clr transformation is not fair in the sense that for the alr transformation additional information in form of an unchanged gene is used. The idea here is of course that it will often be possible to guess such genes correctly.

**Fig. 9:**
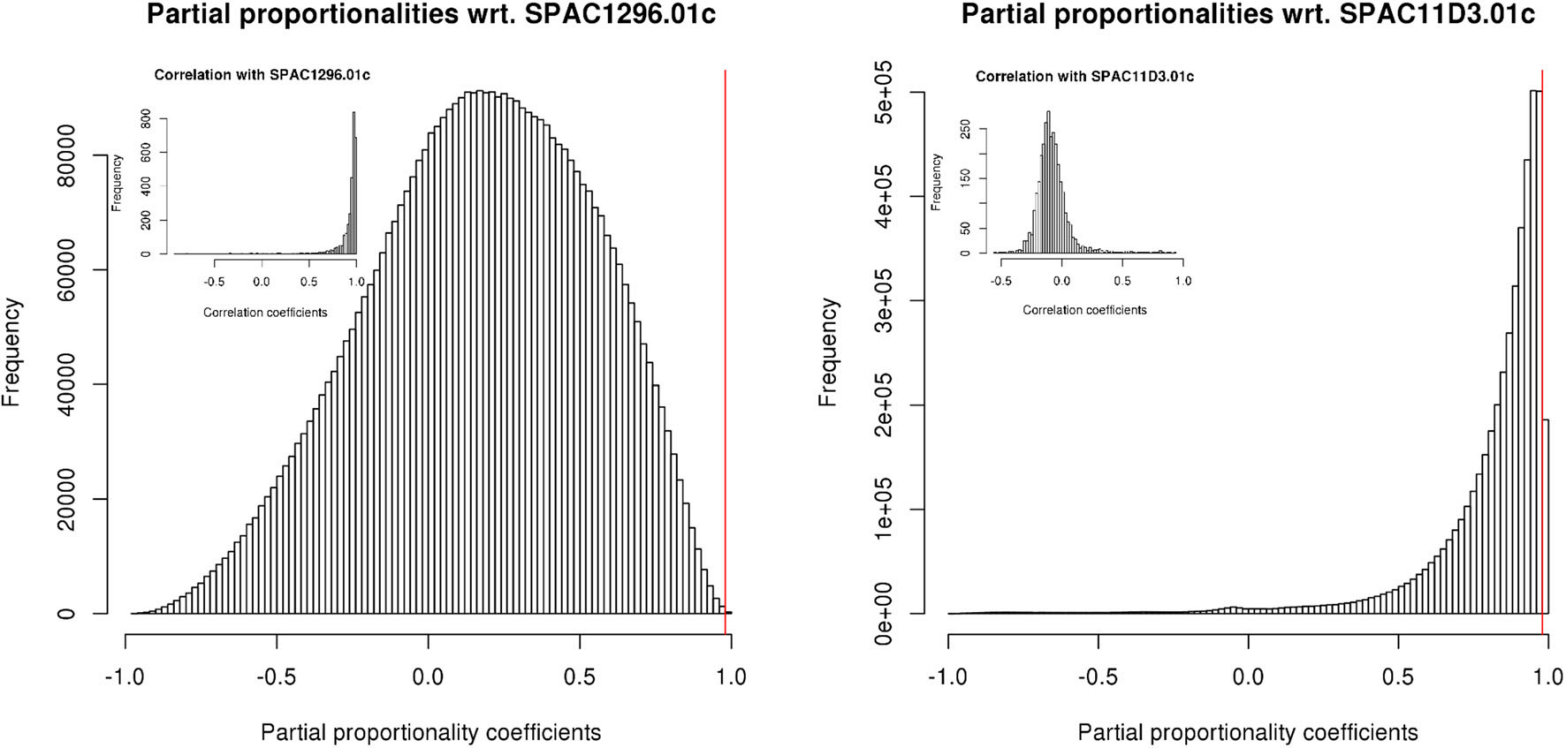
Partial proportionality coefficients on absolute data. Coefficients of all gene pairs with respect to the gene SPAC1296.01c (*left panel*) and the gene SPAC11D3.01c (*right panel*). *Red vertical lines* denote the cut-off of 0.98. Insets show the correlations of the respective gene with all other genes (colour figure online)

Finally, we also calculated partial proportionality coefficients with respect to each of the genes on the absolute data. While our definition of partial proportionality was for the transformed data, where we see its main application, the fact that we do not need a reference on absolute data reduces the number of possible combinations drastically. As an exhaustive study of these coefficients would go beyond the scope of this work, we here limit ourselves to a demonstration that triples of genes exist that lead to partial proportionality. This seems to be the case whenever the gene we are partialling on is behaving “against the trend” of the other genes. In Fig. 9 we show histograms of all partial proportionality coefficients with respect to two genes. The left panel is for a gene that is highly correlated with most genes (see inset) and its partial proportionality coefficients maintain mostly low values. The right panel shows the opposite: a gene with low correlation with most other genes reaches high values of partial proportionality coefficients.

## Discussion

In this contribution we considered log-ratio analysis from the point of view of the absolute data. From this perspective, normalizations can be seen as specific log-ratio transformations allowing to back transform compositional data to absolute data. With this in mind, proportionality coefficients can be applied to traditionally normalized gene expression data and the same conclusions apply without the need for additional data transformations. This holds whenever the normalization procedure preserves ratios between gene expressions, which excludes more invasive procedures like quantile normalizations. Our view point is consistent with an analysis of accuracy of prediction of proportionality comparing results obtained on both types of data. It can also be argued that proportionality is a well-defined concept on relative data in its own right, without the comparison with outcomes on the absolute data. Since the transformed data have no constraints leading to ambiguous results when applying measures like correlation or proportionality, this is a valid point of view that avoids the notion of spurious results. The interpretation of the results is, however, less straightforward and would require the study of the resulting power-law relationships between all the variables including the reference, as can be done for log-ratio correlations Greenacre and Aitchison (2002).

The notion of proportionality was introduced by Lovell et al. to put correlational analysis of relative gene expression data on a more rigorous footing. As shown here, the proposed scaling is not without caveats due to its dependence on the chosen reference. We argue that an educated guess of an approximately unchanged gene or gene set can lead to an alr transformation with great advantages in terms of accuracy. Unfortunately, we will not generally be in a situation where we can evaluate the size and direction of the reference explicitly. Because of this, it is certainly a good idea to simply apply high cut-offs for maximum consistency with absolute outcomes, as suggested by our analytical results.

The proportionality coefficient (a special case of the concordance correlation coefficient) allows for a straight-forward generalization to a notion of partial proportionality, a simple analogy to partial correlations. Here we just derived some expressions for this measure and gave some heuristic interpretations of it. It could be the topic of another contribution to apply all the discussed relationships among genes to build co-expressions networks from pairs (using proportionality and reciprocality), triples (using log-ratio correlations involving a reference), and quadruples of genes (using log-ratio correlations involving the reference and an additional gene, or partial proportionality). This seems an interesting alternative to current approaches based on “crude” correlation and partial correlation.

It is to be wished that the concept of proportionality and log-ratio analysis in general will have a growing impact on established methodology in genome research. Applications range from the calculation of correlation networks Friedman and Alm (2012) over applying log-ratio transformations before doing principal component analysis Greenacre and Aitchison (2002) to alternative normalization procedures of genomic data. Other approaches to correlation on compositional data have been employed, see Filzmoser and Hron (2009) for one involving the isometric log-ratio transformation. Knowledge about the uniformity of expression of particular genes make gene expression data suitable for an alr transformation as argued in the present work. The characteristics of genomic data in general make them a new object of investigation not yet fully explored within the framework of log-ratio analysis. Among these peculiarities are the facts that there are usually many more components than observations and that the data are often counts with various sources of stochasticity. We thus see the necessity for more mathematical and bioinformatic research to fully exploit the strength of the approach.

## Appendix

*Derivation of equation* (1):

13 $$\begin{aligned} \mathrm {var}(A_i-A_j)=\mathrm {var}(A_i)+\mathrm {var}(A_j)-2\mathrm {cov}(A_i,A_j)\nonumber \\ = \mathrm {var}(A_i) \left( 1+\frac{\mathrm {var}(A_j)}{\mathrm {var}(A_i)}-2\sqrt{\frac{\mathrm {var}(A_j)}{\mathrm {var}(A_i)}}\frac{\mathrm {cov}(A_i,A_j)}{\sqrt{\mathrm {var}(A_i)\mathrm {var}(A_j)}}\right) \nonumber \\ =\mathrm {var}(A_i) \left( 1+\beta ^2-2\beta ~r\right) . \end{aligned}$$

### *Proof of Proposition 1*

(i):

14 $$\begin{aligned} \frac{2~\mathrm {cov}(A_i,A_j)}{\mathrm {var}(A_i)+\mathrm {var}(A_j)} =\frac{\mathrm {var}(A_i)+\mathrm {var}(A_j)-\mathrm {var}\left( A_i-A_j\right) }{\mathrm {var}(A_i)+\mathrm {var}(A_j)}\nonumber \\ =1-\frac{\mathrm {var}\left( A_i-A_j\right) }{\mathrm {var}(A_i)+\mathrm {var}(A_j)}. \end{aligned}$$

(ii):

15 $$\begin{aligned} \frac{2~\mathrm {cov}(A_i,A_j)}{\mathrm {var}(A_i)+\mathrm {var}(A_j)}=2~\mathrm {corr}(A_i,A_j)\frac{\sqrt{\mathrm {var}(A_i)\mathrm {var}(A_j)}}{\mathrm {var}(A_i)+\mathrm {var}(A_j)}\nonumber \\ =2r/\frac{\mathrm {var}(A_i)+\mathrm {var}(A_j)}{\sqrt{\mathrm {var}(A_i)\mathrm {var}(A_j)}}=\frac{2r}{\beta +1/\beta }. \end{aligned}$$

(iii): Plugging $\mathrm {var}(A_i+A_j)+\mathrm {var}(A_i-A_j)= 2\left( \mathrm {var}(A_i)+\mathrm {var}(A_j)\right)$ into (i) we find

16 $$\begin{aligned} \frac{2~\mathrm {cov}(A_i,A_j)}{\mathrm {var}(A_i)+\mathrm {var}(A_j)} =1-\frac{2~\mathrm {var}\left( A_i-A_j\right) }{\mathrm {var}(A_i-A_j)+\mathrm {var}(A_i+A_j)}\nonumber \\ =1-\frac{2}{1+1/\tilde{\beta }}=1-\frac{2\tilde{\beta }}{\tilde{\beta }+1}=\frac{\tilde{\beta }+1-2\tilde{\beta }}{\tilde{\beta }+1}. \end{aligned}$$

(iv): We start from

17 $$\begin{aligned} 2~\mathrm {cov}(A_i+A_j,A_i-A_j)=\mathrm {var}(A_i+A_j)+\mathrm {var}(A_i-A_j)-\mathrm {var}\left( A_i+A_j-(A_i-A_j)\right) \nonumber \\ =2~\mathrm {var}(A_i)+2~\mathrm {var}(A_j)-4~\mathrm {var}(A_j)=2~\left( \mathrm {var}(A_i)-\mathrm {var}(A_j)\right) . \end{aligned}$$

Thus with the definition of $\rho$ we find

18 $$\begin{aligned} \frac{2~\mathrm {cov}(A_i+A_j,A_i-A_j)}{\mathrm {var}(A_i+A_j)+\mathrm {var}(A_i-A_j)}=\frac{\mathrm {var}(A_i)-\mathrm {var}(A_j)}{\mathrm {var}(A_i)+\mathrm {var}(A_j)}=\frac{1-\beta ^2}{1+\beta ^2}. \end{aligned}$$

$$\square$$

### *Proof of Proposition 2*

From Proposition 1 (iii), we infer $\rho (A_i,A_j)=(\mathrm {var}(A_i+A_j)-\mathrm {var}(A_i-A_j))/(\mathrm {var}(A_i+A_j)+\mathrm {var}(A_i-A_j)).$ Combining with Eq. (5), we find

19 $$\begin{aligned} \rho (Y_i,Y_j)=\frac{\mathrm {var}(A_i+A_j-2\epsilon )-\mathrm {var}(A_i-A_j)}{\mathrm {var}(A_i+A_j-2\epsilon )+\mathrm {var}(A_i-A_j)}. \end{aligned}$$

The variance involving $\epsilon$ we can now decompose using the first equality in (13):

20 $$\begin{aligned} =\frac{\mathrm {var}(A_i+A_j)-\mathrm {var}(A_i-A_j)+\mathrm {terms}(\epsilon )}{\mathrm {var}(A_i+A_j) +\mathrm {var}(A_i-A_j)+\mathrm {terms}(\epsilon )}, \end{aligned}$$

where $\mathrm {terms}(\epsilon )=\mathrm {var}(2\epsilon )-2\mathrm {cov}(A_i+A_j,2\epsilon )$. Multiplying numerator and denominator by $\mathrm {var}(A_i+A_j)+\mathrm {var}(A_i-A_j)$, we get

21 $$\begin{aligned} =\frac{\mathrm {var}(A_i+A_j)-\mathrm {var}(A_i-A_j)+\mathrm {terms}(\epsilon )}{\mathrm {var}(A_i+A_j)+\mathrm {var}(A_i-A_j)}~\frac{\mathrm {var}(A_i+A_j)+\mathrm {var}(A_i-A_j)}{\mathrm {var}(A_i+A_j)+\mathrm {var}(A_i-A_j)+\mathrm {terms}(\epsilon )}\nonumber \\ =\left( \rho (A_i,A_j)+\frac{\mathrm {terms}(\epsilon )}{\mathrm {var}(A_i+A_j)+\mathrm {var}(A_i-A_j)}\right) \left( \frac{1}{1+\frac{\mathrm {terms}(\epsilon )}{\mathrm {var}(A_i+A_j)+\mathrm {var}(A_i-A_j)}}\right) . \end{aligned}$$

Let us use the shorthand $g(\epsilon )=\frac{\mathrm {terms}(\epsilon )}{\mathrm {var}(A_i+A_j)+\mathrm {var}(A_i-A_j)}$. We can expand the term in the second pair of brackets:

22 $$\begin{aligned} \rho (Y_i,Y_j)=\left( \rho (A_i,A_j)+g(\epsilon )\right) \left( 1-g(\epsilon )+g^2(\epsilon )-\cdots \right) \nonumber \\ =\rho (A_i,A_j)+(1-\rho (A_i,A_j))g(\epsilon )-(1-\rho (A_i,A_j))g^2(\epsilon )+\cdots \end{aligned}$$

Let us now evaluate $g(\epsilon )$. We have

23 $$\begin{aligned} g(\epsilon )=\frac{\mathrm {var}(2\epsilon )-2\mathrm {cov}(A_i+A_j,2\epsilon )}{\mathrm {var}(A_i+A_j)+\mathrm {var}(A_i-A_j)}=\frac{\mathrm {var}(2\epsilon )-2\mathrm {cov}(A_i+A_j,2\epsilon )}{2\left( \mathrm {var}(A_i)+\mathrm {var}(A_j)\right) }. \end{aligned}$$

The covariance term can be expressed in terms of correlation:

24 $$\begin{aligned} \frac{\mathrm {cov}(A_i+A_j,2\epsilon )}{\mathrm {var}(A_i)+\mathrm {var}(A_j)}=\frac{\mathrm {cov}(A_i+A_j,2\epsilon )}{\sqrt{\mathrm {var}(A_i+A_j)\mathrm {var}(2\epsilon )}}~\frac{\sqrt{\mathrm {var}(A_i+A_j)\mathrm {var}(2\epsilon )}}{\mathrm {var}(A_i)+\mathrm {var}(A_j)}\nonumber \\ =\mathrm {corr}(A_i+A_j,2\epsilon )\sqrt{1+\rho (A_i,A_j)}\sqrt{\frac{\mathrm {var}(2\epsilon )}{\mathrm {var}(A_i)+\mathrm {var}(A_j)}}, \end{aligned}$$

where we used the fact that $\mathrm {var}(A_i+A_j)/(\mathrm {var}(A_i)+\mathrm {var}(A_j))=1+\rho (A_i,A_j)$. Plugging this into (23) and using the notation $\varphi (\epsilon )$ defined in the proposition, we find

25 $$\begin{aligned} g(\epsilon )=\frac{\varphi ^2(\epsilon )}{2}-\sqrt{1+\rho (A_i,A_j)}~\mathrm {corr} (A_i+A_j,2\epsilon )~\varphi (\epsilon ). \end{aligned}$$

Let us now return to (22). With the new expression for $g(\epsilon )$ we find

26 $$\begin{aligned} \frac{\rho (Y_i,Y_j)-\rho (A_i,A_j)}{1-\rho (A_i,A_j)}= \frac{\varphi ^2(\epsilon )}{2}-\sqrt{1+\rho (A_i,A_j)}\mathrm {corr}(A_i+A_j,2\epsilon )\varphi (\epsilon )-\nonumber \\ \left( \frac{\varphi ^2(\epsilon )}{2}-\sqrt{1+\rho (A_i,A_j)}\mathrm {corr}(A_i+A_j,2\epsilon )\varphi (\epsilon )\right) ^2+\cdots \nonumber \\ =-\sqrt{1+\rho (A_i,A_j)}\mathrm {corr}(A_i+A_j,2\epsilon )\varphi (\epsilon )+\nonumber \\ \left( \frac{1}{2}-\left( 1+\rho (A_i,A_j)\right) \mathrm {corr}^2(A_i+A_j,2\epsilon )\right) \varphi ^2(\epsilon )+\cdots \end{aligned}$$

$$\square$$

### *Proof of Proposition 3*

Using the definition of $\tilde{\beta }$ of Proposition 1 (iii), for the relative data we define

27 $$\begin{aligned} \tilde{\beta }_Y:=\sqrt{\frac{\mathrm {var}(Y_i-Y_j)}{\mathrm {var}(Y_i+Y_j)}}= \sqrt{\frac{\mathrm {var}(A_i-A_j)}{\mathrm {var}(A_i+A_j-2A_d)}}, \end{aligned}$$

where the equality comes from Eq. (5). We now find

28 $$\begin{aligned} \tilde{\beta }_Y^2 &=\frac{\mathrm {var}(A_i-A_j)}{\mathrm {var}(A_i+A_j)+\mathrm {terms}(A_d)}\nonumber \\ &=\frac{\mathrm {var}(A_i-A_j)}{\mathrm {var}(A_i+A_j)}~\frac{\mathrm {var}(A_i+A_j)}{\mathrm {var}(A_i+A_j)+\mathrm {terms}(A_d)}. \end{aligned}$$

where we used the shorthand terms$(A_d)=$var$(2A_d)-2$cov$(A_i+A_j,2A_d)$. We thus find

29 $$\begin{aligned} \tilde{\beta }_Y^2 &=\tilde{\beta }^2\left( \frac{\mathrm {var}(A_i+A_j)}{\mathrm {var}(A_i+A_j)+\mathrm {terms}(A_d)}\right) \nonumber \\ &=\tilde{\beta }^2\left( \frac{1+\rho }{1+\rho +2g(A_d)}\right) , \end{aligned}$$

where the numerator and denominator were divided by $\mathrm {var}(A_i)+\mathrm {var}(A_j)$ and the definition of $g(A_d)$ (c.f. Eq. (23)) was used. From Proposition 1 (iii) it now follows that

30 $$\begin{aligned} \rho (Y_i,Y_j)-\rho (A_i,A_j)=\frac{1-\tilde{\beta }_Y^2}{1+\tilde{\beta }_Y^2}-\frac{1-\tilde{\beta }^2}{1+\tilde{\beta }^2}=\frac{2(\tilde{\beta }^2-\tilde{\beta }_Y^2)}{(1+\tilde{\beta }^2)(1+\tilde{\beta }_Y^2)}. \end{aligned}$$

Filling in (29), we obtain

31 $$\begin{aligned} \rho (Y_i,Y_j)-\rho (A_i,A_j)=\frac{2\tilde{\beta }^2\left( 1-\frac{1+\rho }{1+\rho +2g(A_d)}\right) }{(1+\tilde{\beta }^2)\left( 1+\tilde{\beta }^2\frac{1+\rho }{1+\rho +2g(A_d)}\right) }\nonumber \\ =\frac{2\tilde{\beta }^2}{1+\tilde{\beta }^2}~\frac{2g(A_d)}{(1+\rho )(1+\tilde{\beta }^2)+2g(A_d)} \end{aligned}$$

The prefactor involving $\tilde{\beta }^2$ evaluates to $1-\rho$, and $1+\tilde{\beta }^2=2/(1+\rho )$. With this we finally find

32 $$\begin{aligned} \frac{\rho (Y_i,Y_j)-\rho (A_i,A_j)}{1-\rho (A_i,A_j)}=\frac{2g(A_d)}{2g(A_d)+2}=\frac{1}{1+1/g(A_d)}. \end{aligned}$$

It remains to show that $g(A_d)=F(A_d)/2$ defined in the proposition. This follows from (25). $\square$

### **Lemma 1**

(i) $F(A_d)$ as a function of $\varphi$ takes its minimum value $F_\mathrm {min}=-(1+\rho (A_i,A_j))\mathrm {corr}^2(A_i+A_j,A_d)$ at $\varphi _\mathrm {min}=\sqrt{1+\rho (A_i,A_j)}\mathrm {corr}(A_i+A_j,A_d)$.
(ii) Let $\Delta \rho :=\rho (Y_i,Y_j)-\rho (A_i,A_j)$. Then $F(A_d)\ge 0\Longleftrightarrow \Delta \rho \ge 0$.
(iii) $\Delta \rho$ as a function of *F* is monotonically increasing and thus takes its minimum value at $F_\mathrm {min}$.

### *Proof of Lemma 1*

(i): With the definition of $F(A_d)$ in Proposition 3, we find

33 $$\begin{aligned} F^\prime (\varphi )=2\varphi -2\sqrt{1+\rho (A_i,A_j)}\mathrm {corr}(A_i+A_j,A_d). \end{aligned}$$

As the second derivative evaluates to $2>0$, setting (33) to 0 implies a minimum at $\varphi _\mathrm {min}=\sqrt{1+\rho (A_i,A_j)}\mathrm {corr}(A_i+A_j,A_d)$. Putting this into F yields the value of $F_\mathrm {min}$.

(ii): $F(A_d)\ge 0\Rightarrow \Delta \rho \ge 0$ is trivial as $\rho \le 1$.

$\Delta \rho \ge 0\Rightarrow F(A_d)\ge 0$: From Proposition 3 it follows that $\Delta \rho =(1-\rho )/(1+2/F)$. $\Delta \rho >0$ leads to $1+2/F\ge 0$. The only way this can be true for negative *F* is for $F<-2$. But we know from Lemma 1 (i) that $F\ge -(1+\rho )\mathrm {corr}^2(A_i+A_j,A_d)\ge -2$, so $F\ge 0$.

(iii): We have

34 $$\begin{aligned} \Delta \rho ^\prime (F)=-\frac{1-\rho }{\left( 1+2/F\right) ^2}\frac{-2}{F^2}=\frac{2(1-\rho )}{(F+2)^2}\ge 0. \end{aligned}$$

$$\square$$

### *Proof of Corollary 1*

From Lemma 1 (ii) we know that $$\Delta \rho \ge 0$$ implies $$F(A_d)\ge 0$$, so we only need to show that $${\text{corr}}(A_i+A_j,A_d)\le \sqrt{\frac{\mathrm {var}(A_d)}{\mathrm {var}(A_i+A_j)}}$$ implies $$F(A_d)\ge 0$$. We start with $$F(A_d)\ge 0$$: From $$\varphi ^2-2\sqrt{1+\rho }$$$${\text{corr}}(A_i+A_j,A_d)\varphi \ge 0$$ it follows that

35 $$\begin{aligned} \varphi \ge 2\sqrt{1+\rho }~\mathrm {corr}(A_i+A_j,A_d). \end{aligned}$$

Now we use

36 $$\begin{aligned} \varphi =\sqrt{\frac{\mathrm {var}(2A_d)}{\mathrm {var}(A_i)+\mathrm {var}(A_j)}}=\sqrt{\frac{4\mathrm {var}(A_d)}{\mathrm {var}(A_i)+\mathrm {var}(A_j)}}=2\sqrt{\frac{\mathrm {var}(A_d)(1+\rho )}{\mathrm {var}(A_i+A_j)}}, \end{aligned}$$

where the last equality comes from the fact that var$$(A_i+A_j)/\left( \mathrm {var}(A_i)+\mathrm {var}(A_j)\right) =1+\rho.$$ Putting this into Eq. (35), we conclude $$\sqrt{\frac{\mathrm {var}(A_d)}{\mathrm {var}(A_i+A_j)}}\,\ge$$$$\text{corr}(A_i+A_j,A_d).$$ For the other direction of the proof we assume corr$$(A_i+A_j,A_d)\le \sqrt{\frac{\mathrm {var}(A_d)}{\mathrm {var}(A_i+A_j)}}$$: Let us use (36) to infer that

37 $$\begin{aligned} \frac{\varphi }{2\sqrt{1+\rho }}\ge \mathrm {corr}(A_i+A_j,A_d). \end{aligned}$$

From this it follows that 2$\sqrt{1+\rho }$ corr($A_i+A_j,A_d)\le \varphi$, comparing with (35), we conclude $F\ge 0$. $\square$

### *Proof of Corollary 2*

Let us look at the situation where on the relative data we equal the cut-off *K*: Inserting into $\rho (Y_i,Y_j)=\rho (A_i,A_j)+\Delta \rho =K$ the expression for $\Delta \rho$, we obtain $(1-\rho (A_i,A_j))/(1+2/F)=K-\rho (A_i,A_j)$, or $(1-\rho )/(K-\rho )-1=(1-\rho -K+\rho )/(K-\rho )=(1-K)/(K-\rho )=2/F$. So the value for *F* when $\rho (Y_i,Y_j)$ just equals the cut-off is

38 $$\begin{aligned} F=2\frac{K-\rho (A_i,A_j)}{1-K}=\tilde{K}. \end{aligned}$$

Now we know from Lemma 1 (iii) that $F\ge \tilde{K}$ implies $\rho (Y_i,Y,j)\ge K$ and $F<\tilde{K}$ implies $\rho (Y_i,Y,j)<K$. From (38) we also see that $\tilde{K}\le 0$ implies $\rho (A_i,A_j)\ge K$ and $\tilde{K}>0$ implies $\rho (A_i,A_j)<K$. Comparing these statements with the definitions of $\mathcal {S}_\mathrm {TP}$, $\mathcal {S}_\mathrm {FP}$, $\mathcal {S}_\mathrm {TN}$ and $\mathcal {S}_\mathrm {FN}$ completes the proof. $\square$

### *Proof of Corollary 3*

From the proof of Corollary 2 we know that $\rho (Y_i,Y_j)\ge K$ iff $F(A_d)\ge \tilde{K}$. To show the latter, we solve $F(A_d)-\tilde{K}=0$. We obtain

39 $$\begin{aligned} \varphi _\pm =-p/2\pm \sqrt{p^2/4+\tilde{K}}, \end{aligned}$$

where we used the shorthand $p:=-2\sqrt{1+\rho }$ corr$(A_i+A_j,A_d)$. As $\tilde{K}>0$ (following from $\rho (A_i,A_j)<K$), only $\varphi _+$ is positive. To show that $\varphi >\varphi _+$ yields $F>\tilde{K}$, we insert $\varphi _+$ into the derivative of *F* given in (33):

40 $$\begin{aligned} F^\prime (\varphi _+)=-p+\sqrt{p^2/4+\tilde{K}}+p=\sqrt{p^2/4+\tilde{K}}>0. \end{aligned}$$

We conclude that $F(A_d)>\tilde{K}$ for $\varphi >\varphi _+$. Let us now bound $\varphi _+$ from above. Let us start with the expression under the root:

41 $$\begin{aligned} p^2/4+\tilde{K}=(1+\rho )\mathrm {corr}^2(A_i+A_j,A_d)+\frac{2(K-\rho )}{1-K}\le \frac{(1+\rho )(1-K)+2(K-\rho )}{1-K}\nonumber \\ =\frac{1+\rho -K-K\rho +2K-2\rho }{1-K}=\frac{(1+K)(1-\rho )}{1-K}\le \frac{4}{1-K}. \end{aligned}$$

We can thus bound $\varphi _+$ by

42 $$\begin{aligned} \varphi _+=-p/2+\sqrt{p^2/4+\tilde{K}}\le \sqrt{2}+\frac{2}{\sqrt{1-K}} =\frac{\sqrt{2(1-K)}+2}{\sqrt{1-K}}\le \frac{4}{\sqrt{1-K}}. \end{aligned}$$

According to the assumption, for genes from the unchanged set we have

43 $$\begin{aligned} \varphi (A_d)=\sqrt{\frac{\mathrm {var}(2A_d)}{\mathrm {var}(A_i)+\mathrm {var}(A_j)}} \ge \sqrt{\frac{\mathrm {var}(2A_d)}{2\mathrm {var}(\epsilon )}}=\sqrt{\frac{2\mathrm {var}(A_d)}{\mathrm {var}(\epsilon )}}. \end{aligned}$$

Comparing with (42), we see that to exceed the value of $\varphi _+$ it is sufficient to have

44 $$\begin{aligned} \sqrt{\frac{2\mathrm {var}(A_d)}{\mathrm {var}(\epsilon )}}\ge \frac{4}{\sqrt{1-K}}. \end{aligned}$$

Squaring and rearranging, this proves the corollary. $\square$

### **Lemma 2**

*For scalar random variables*$Y_i, Y_k~\,and~\,\hat{Y}_i$*the linear least-squares predictor of*$Y_i$*wrt.*$Y_k,$*we have*

(i) $$\mathrm {var}(Y_i-\hat{Y}_i)=\mathrm {var}(Y_i)-\mathrm {var}(\hat{Y}_i$$),
(ii) $$\mathrm {var}(\hat{Y}_i)=\mathrm {cov}^2(Y_i,Y_k)/\mathrm {var}(Y_k)$$.

### *Proof*

see e.g. Whittaker (2008), chapter 5. $\square$

### *Proof of Proposition 4*

(i): We start from the expression for $\rho$ in Proposition 1 (i). Since it contains three similar variance terms, it suffices to show that

45 $$\begin{aligned} \mathrm {var}(Y_i-\hat{Y}_i)=\mathrm {var}(Y_i)\left( 1-\mathrm {corr}^2(Y_i,Y_k)\right) , \end{aligned}$$

and then substitute $Y_j$ and $Y_i-Y_j$ for $Y_i$ (linearity of the predictor implies $\widehat{Y_i-Y_j}=\hat{Y}_i-\hat{Y}_j$). We now have

46 $$\begin{aligned} \mathrm {var}(Y_i-\hat{Y}_i)=\mathrm {var}(Y_i)-\frac{\mathrm {cov}^2(Y_i,Y_k)}{\mathrm {var}(Y_k)}=\mathrm {var}(Y_i)\left( 1-\mathrm {corr}^2(Y_i,Y_k)\right) , \end{aligned}$$

where the first equality comes from combining the statements in Lemma 2 and the second one from the definition of the correlation coefficient. This proves the first identity of the proposition.

(ii): For the second identity, we use the well-known formula for the partial correlation

47 $$\begin{aligned} \mathrm {corr}(Y_i-\hat{Y}_i,Y_j-\hat{Y}_j)=\frac{\mathrm {corr}(Y_i,Y_j)-\mathrm {corr}(Y_i,Y_k) \mathrm {corr}(Y_j,Y_k)}{\sqrt{(1-\mathrm {corr}^2(Y_i,Y_k))(1-\mathrm {corr}^2(Y_j,Y_k))}}. \end{aligned}$$

(See e.g. Whittaker (2008).) Combining with the expression for $\rho$ in Proposition 1 (ii), we have

48 $$\begin{aligned}&\mathrm {prop}(Y_i-\hat{Y}_i,Y_j-\hat{Y}_j)=\frac{2~\mathrm {corr}(Y_i-\hat{Y}_i,Y_j-\hat{Y}_j)}{\sqrt{\frac{\mathrm {var}(Y_j-\hat{Y}_j)}{\mathrm {var}(Y_i-\hat{Y}_i)}}+\sqrt{\frac{\mathrm {var}(Y_i-\hat{Y}_i)}{\mathrm {var}(Y_j-\hat{Y}_j)}}}\nonumber \\&=\frac{\mathrm {corr}(Y_i,Y_j)-\mathrm {corr}(Y_i,Y_k)\mathrm {corr}(Y_j,Y_k)}{\sqrt{(1-\mathrm {corr}^2(Y_i,Y_k))(1-\mathrm {corr}^2(Y_j,Y_k))}}\frac{2}{\sqrt{\frac{\mathrm {var}(Y_j)(1-\mathrm {corr}^2(Y_j,Y_k))}{\mathrm {var}(Y_i)(1-\mathrm {corr}^2(Y_i,Y_k))}}+\sqrt{\frac{\mathrm {var}(Y_i)(1-\mathrm {corr}^2(Y_i,Y_k))}{\mathrm {var}(Y_j)(1-\mathrm {corr}^2(Y_j,Y_k))}}}\nonumber \\&=\frac{2\left( \mathrm {corr}(Y_i,Y_j)-\mathrm {corr}(Y_i,Y_k)\mathrm {corr}(Y_j,Y_k)\right) }{(1-\mathrm {corr}^2(Y_j,Y_k))\sqrt{\frac{\mathrm {var}(Y_j)}{\mathrm {var}(Y_i)}}+(1-\mathrm {corr}^2(Y_i,Y_k))\sqrt{\frac{\mathrm {var}(Y_i)}{\mathrm {var}(Y_j)}}}. \end{aligned}$$

(iii): In the expression for $\rho$ of Proposition 1 (iii), plugging the new variables into $\tilde{\beta }^2$ evaluates to

49 $$\begin{aligned} \frac{\mathrm {var}(Y_i-\hat{Y}_i-(Y_j-\hat{Y}_j))}{\mathrm {var}(Y_i-\hat{Y}_i+Y_j-\hat{Y}_j)}=\frac{\mathrm {var}(Y_i-Y_j-\widehat{(Y_i-Y_j)})}{\mathrm {var}(Y_i+Y_j-\widehat{(Y_i+Y_j)})}\nonumber \\ =\frac{\mathrm {var}(Y_i-Y_j)\left( 1-\mathrm {corr}^2(Y_i-Y_j,Y_k)\right) }{\mathrm {var}(Y_i+Y_j)\left( 1-\mathrm {corr}^2(Y_i+Y_j,Y_k)\right) }=\frac{\tilde{\beta }^2}{G}, \end{aligned}$$

where the first identity follows from linearity of the predictor, the second one from (46), and the third one from the definitions of $\tilde{\beta }$ and *G*. $\square$

## Footnotes

1 Note that we slightly differ from the expression in Lovell et al. (2015) in that we define $\beta$ without a sign.

2 Note that there is debate about whether such correlations should be called spurious because they can be interpreted correctly avoiding an over-simplified interpretation based on the correlation coefficient and considering the correct regression model, see the discussion in Firebaugh and Gibbs (1985)
